# Use of CRISPR/Cas9-edited HEK293 cells reveals that both conventional and novel protein kinase C isozymes are involved in mGlu_5a_ receptor internalization

**DOI:** 10.1016/j.jbc.2022.102466

**Published:** 2022-09-08

**Authors:** Jeffrey R. van Senten, Thor C. Møller, Ee Von Moo, Sofie D. Seiersen, Hans Bräuner-Osborne

**Affiliations:** Department of Drug Design and Pharmacology, University of Copenhagen, Copenhagen, Denmark

**Keywords:** mGlu5, metabotropic glutamate receptor, G protein-coupled receptor, receptor endocytosis, receptor internalization, receptor regulation, PKC, CRISPR/cas, aPKC, atypical PKC, BRET, bioluminescence resonance energy transfer, cPKC, conventional PKC, DAG, diacylglycerol, dFBS, dialyzed fetal bovine serum, DPBS, Dulbecco’s phosphate buffered saline, GPCR, G protein-coupled receptor, GRK, GPCR kinase, HBSS, Hank’s balanced salt solution, IP_1_, inositol monophosphate 1, mGlu_5_, metabotropic glutamate receptor 5, nPKC, novel PKC, PDBu, phorbol 12,13-dibutyrate, qPCR, quantitative PCR, RACK, receptors for activated C kinase

## Abstract

The internalization of G protein–coupled receptors (GPCRs) can be regulated by PKC. However, most tools available to study the contribution of PKC isozymes have considerable limitations, including a lack of selectivity. In this study, we generated and characterized human embryonic kidney 293A (HEK293A) cell lines devoid of conventional or novel PKC isozymes (ΔcPKC and ΔnPKC) and employ these to investigate the contribution of PKC isozymes in the internalization of the metabotropic glutamate receptor 5 (mGlu_5_). Direct activation of PKC and mutation of rat mGlu_5a_ Ser^901^, a PKC-dependent phosphorylation site in the receptor C-tail, both showed that PKC isozymes facilitate approximately 40% of the receptor internalization. Nonetheless, we determined that mGlu_5a_ internalization was not altered upon the loss of cPKCs or nPKCs. This indicates that isozymes from both classes are involved, compensate for the absence of the other class, and thus fulfill dispensable functions. Additionally, using the Gαq/11 inhibitor YM-254890, GPCR kinase 2 and 3 (GRK2 and GRK3) KO cells, and a receptor containing a mutated putative adaptor protein complex 2 (AP-2) interaction motif, we demonstrate that internalization of rat mGlu_5a_ is mediated by Gαq/11 proteins (77% of the response), GRK2 (27%), and AP-2 (29%), but not GRK3. Our PKC KO cell lines expand the repertoire of KO HEK293A cell lines available to research GPCR pharmacology. Moreover, since pharmacological tools to study PKC isozymes generally lack specificity and/or potency, we present the PKC KO cell lines as more specific research tools to investigate PKC-mediated aspects of cell biology.

G protein-coupled receptor (GPCR) function, signaling, and regulation are complex molecular processes. They involve many different components, intricate networks, as well as feedback mechanisms (1, 2, 3). CRISPR/Cas-mediated genome editing is a valuable addition to pharmacological research and offers new opportunities to study GPCR biology (4). Knock-in of protein tags or mutations facilitate the study of protein function at native expression levels (5, 6), whereas cell lines devoid of receptors (7, 8), G proteins (9, 10), GPCR kinases (GRKs) (11, 12), PKA (13), or arrestins (11, 14, 15) provide new model systems to study the contribution of specific proteins or protein classes. In addition to GRKs and PKAs, the second messenger–sensitive PKC family is also known to regulate GPCRs in terms of desensitization and internalization and to propagate signaling downstream of GPCRs (16). Besides PKCα (17), however, PKC-null cell lines are lacking from the research toolkit.

The PKC protein family of serine/threonine-specific kinases is involved in signal transduction of extracellular stimuli and consists of 10 isozymes subdivided into conventional PKCs (cPKC), novel PKCs (nPKC), and atypical PKCs (aPKC) (18, 19). Since their catalytic domains, containing ATP and substrate-binding regions, are highly homologous, the division is based on variation in the regulatory domains that control enzyme activity. Translocation of cPKC (PKCα, βI, βII, and γ) isozymes to cellular membranes is regulated by Ca^2+^ and diacylglycerol (DAG) binding to their C2 and C1 domain, respectively. nPKCs (PKCδ, ε, η, and θ) contain a nonfunctional C2 domain, rendering them insensitive to Ca^2+^, and a C1 domain that has two orders of magnitude higher affinity for DAG than that of cPKCs (20). aPKC isoforms (PKCζ and λ/ι) are insensitive to second messengers due to a nonfunctional C1 domain and the absence of a C2 domain and instead rely on protein–protein interactions via the Phox and Bem 1 (PB1) domain for their activation (21). Depending on the isozyme, additional C1 and C2 domain-interacting lipid second messengers have been reported to modulate PKC function (22). A shared moiety in the regulatory domain of all PKC isozymes is the autoinhibitory pseudosubstrate, which sterically blocks substrate binding to the catalytic site. Upon second messenger binding, the pseudosubstrate is allosterically released allowing enzymatic activity (23). Substrate sequence recognition is largely conserved among PKC isozymes (24) and hence substrate specificity is mainly regulated by protein–protein interactions, localizing enzymes in the vicinity of their substrates (25). Furthermore, consensus phosphorylation sites of several other kinases, including calcium/calmodulin-dependent protein kinase II (CaMKII), PKA, and Rho-associated protein kinase (Rock), are similar to that of PKC, indicating shared substrates and convergence of signaling pathways (24). Since pharmacological tools to study the PKC family generally lack specificity and/or potency, hampering delineation of the role of individual PKC isozymes (26), genetic approaches are promising alternatives.

Metabotropic glutamate receptor 5 (mGlu_5_) is widely expressed at postsynaptic membranes in the central nervous system where it contributes to neuronal development and synaptic plasticity upon activation by the excitatory neurotransmitter L-glutamate (27). As a GPCR primarily coupled to Gα_q/11_ proteins, mGlu_5_ mediates PKC activation through phospholipase C β–driven production of DAG and mobilization of Ca^2+^. In turn, PKC regulates several aspects of receptor function by phosphorylation of intracellular mGlu_5_ domains. Stimulation of mGlu_5_ leads to single Ca^2+^ transients or characteristic oscillatory Ca^2+^ signaling. Ca^2+^ oscillations require repeated transient PKC-mediated phosphorylation of the rat mGlu_5a_ receptor at Ser^839^ (28, 29). Interestingly, responsiveness upon repeated stimulation is preserved in oscillating cells, in contrast to the single Ca^2+^ transients that are subject of desensitization (28). The conditions favoring and the significance of differential Ca^2+^ signals are not yet fully understood. However, Ser^839^ in rat mGlu_5a_ is part of the Gα_q/11_-binding domain in the C terminal tail of the receptor, which might indicate modulation of G protein coupling. Phosphorylation by PKC at Ser^901^ regulates the endocytosis of rat mGlu_5a_ by impairing calmodulin binding (30). Due to overlapping binding sites, reduced calmodulin occupancy of the receptor facilitates the interaction with E3 ligase seven in absentia homolog 1A (Siah-1A), which mediates mGlu_5_ endocytosis and sorting for lysosomal degradation through receptor ubiquitination (31, 32). Using peptides inhibiting or stimulating PKCε membrane translocation, agonist-induced mGlu_5_ internalization in rat nucleus accumbens was shown to be mediated by PKCε (33).

Here, we set out to investigate the role of PKC isozymes in the regulation of GPCRs. To this end, we generated and characterized cPKC KO (ΔcPKC) and nPKC KO (ΔnPKC) human embryonic kidney 293A (HEK293A) cell lines. As a case study, we studied mGlu_5a_ internalization and show this to be driven by Gα_q/11_, GRK2, PKC, and adaptor protein 2 (AP-2), while being independent of GRK3. Using the PKC KO cells, we show functional redundancy of second messenger–sensitive PKCs in regulation of mGlu_5a_ function at the level of receptor internalization. In a broader perspective, we envision that the HEK293A ΔcPKC and ΔnPKC cell lines will be valuable resources to study PKC-mediated molecular mechanisms, such as receptor regulation and cellular signaling.

## Results

### Gα_q/11_, PKC, GRK2, and AP-2 contribute to mGlu_5a_ internalization

**Figure 1:**
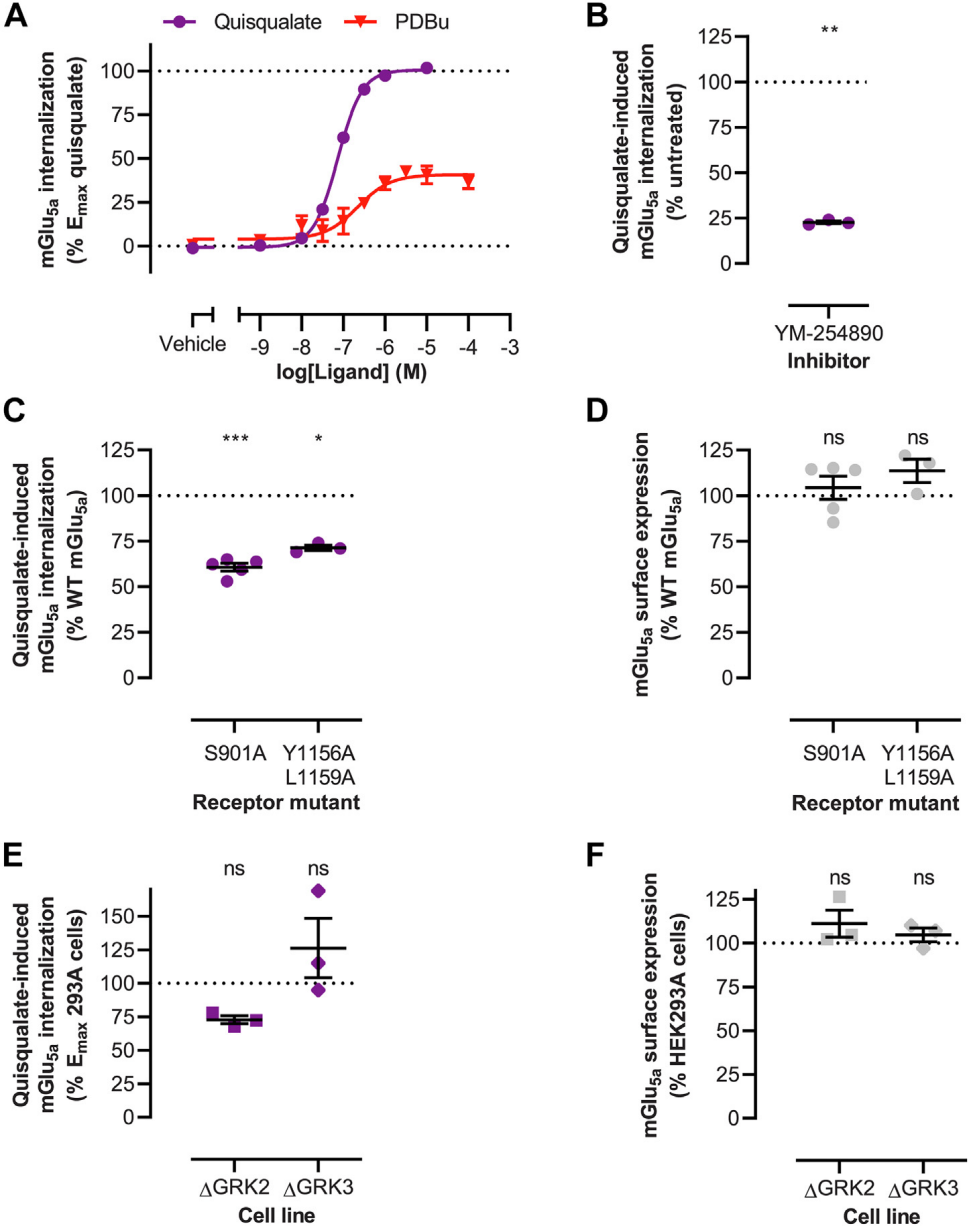
**Delineating mGlu**_**5a**_**receptor internalization.***A*, mGlu_5a_ receptor internalization upon receptor stimulation with quisqualate or PKC stimulation with PDBu. Internalization was measured over a 120-min period following agonist addition. Agonists were present during the internalization measurements. *B*, inhibition of mGlu_5a_ receptor internalization stimulated by 10 μM quisqualate upon a 30-min preincubation with 1 μM Gα_q/11_ protein inhibitor YM-254890. Internalization was measured over a 66-min period following quisqualate addition. *C*, internalization of mGlu_5a_ receptor Ser^901^Ala (S901A) and Tyr^1156^Ala/Lys^1159^Ala (Y1156A, L1159A) mutants in response to 10 μM quisqualate and (*D*) their surface expression compared to WT receptor. Internalization was measured over a 66-min period following quisqualate addition. *E*, internalization of mGlu_5a_ in response to 10 μM quisqualate and (*F*) receptor surface expression in HEK293A cells devoid of GRK2 or GRK3, as compared to parental HEK293A cells. Internalization was measured over a 66-min period following quisqualate addition. Data represent mean and S.E.M. of the area under the curve of real-time internalization measurements over a 0 to 120 min (A) or 0 to 66 min (B + C + E) time interval following agonist addition in HEK293A cells (Fig. S1) after subtraction of the buffer responses followed by normalization to receptor agonist response from three (B + C(Y1156A, L1159A)+E), four (A), or five (C(S901A)) independent experiments performed in triplicate. Data represent mean and S.E.M. from three (D(Y1156A, L1159A)+F) or five (D(S901A)) independent experiments performed in triplicate. Error bars not shown lie within the dimension of the symbol. ns *p* ≥ 0.05, ∗*p* < 0.05, ∗∗*p* < 0.01, ∗∗∗*p* < 0.001, as determined using paired *t* test (B) or one-way ANOVA with Holm-Sidak’s multiple comparison test (*C*–*F*) on raw data with WT receptor (C + D) as untreated condition (*B*) or HEK293A cells (E + F) as control condition. mGlu5, metabotropic glutamate receptor 5; PDBu, phorbol 12,13-dibutyrate.

To investigate the mechanism of mGlu_5a_ internalization, we employed a time-resolved FRET–based real-time internalization assay. In this assay, SNAP-tagged cell surface receptors were covalently labeled with a long-lifetime FRET donor and exposed to assay buffer containing a cell-impermeable FRET acceptor (34, 35). Receptor internalization results in a loss of energy transfer, due to separation of the donor and acceptor molecules. To prevent mGlu_5a_ activation by cellular-released glutamate (36), HEK293A cells were cotransfected with the high affinity glutamate transporter excitatory amino acid transporter 3 (EAAT3) (37). Stimulation of mGlu_5a_ with the surrogate agonist quisqualate, which is not a substrate of EAAT3 (38), induced concentration-dependent receptor internalization (Figs. 1*A*, S1 and Table 1). Heterologous receptor internalization by second messenger–dependent kinases does not require active receptor conformations, and direct activation of PKC using DAG-mimetic phorbol 12,13-dibutyrate (PDBu) indeed induced mGlu_5a_ internalization with up to 40% efficacy compared to quisqualate. Inhibition of Gα_q/11_ protein using YM-254890 inhibited the internalization of mGlu_5a_ by 77%, indicating that receptor internalization is largely Gα_q/11_ mediated (Fig. 1*B*). Importantly, YM-254890 showed no interference with the donor FRET signal (data not shown), demonstrating specific inhibition of mGlu_5a_ internalization. The contribution of PKC was further probed via mutagenesis of the mGlu_5a_ Ser^901^ PKC target residue. At expression levels similar to WT receptor, quisqualate-stimulated endocytosis was reduced by 39% for mGlu_5a_ Ser^901^Ala (Fig. 1, *C* and *D*). This correlates well to the degree of receptor internalization in response to PDBu-driven PKC activation. Unfortunately, several bisindolylmaleimide-based small molecule PKC inhibitors (Gö6983, Ro-32-0432 and BisIV) could not be used in the internalization assay due to interference of those molecules with the donor FRET signal (data not shown). Additionally, we used our previously reported KO cell lines to assess the contribution of GRK2 and GRK3 (11). Albeit not a statistically significant reduction, the absence of GRK2 reduced receptor internalization by 27%, at receptor cell surface expression similar to HEK293A cells (Fig. 1, *E* and *F*). In contrast, mGlu_5a_ internalization was not impaired upon the loss of GRK3 (Fig. 1, *E* and *F*). The C-terminal tail of mGlu_5a_ furthermore contains a putative Y-X-X-L interaction motif for AP-2 (39), through which AP-2 could facilitate endocytosis of mGlu_5a_ via clathrin-coated vesicles. Mutation of the AP-2–binding motif (rat mGlu_5a_ Tyr^1156^Ala/Lys^1159^Ala) reduced agonist-induced receptor internalization by 29%, while the receptor expression was not altered (Fig. 1, *C* and *D*). Altogether, these data indicate that mGlu_5a_ internalization is partially mediated by Gα_q/11_, PKC isozymes, GRK2 and AP-2, but not GRK3.

**Table 1:** mGlu_5a_ internalization in HEK293A cells

Ligand	Quisqualate	PDBu
pEC_50_		
Mean	7.14	6.74
S.E.M.	0.02	0.20
E_max_ (%)		
Mean	100	39
S.E.M.	−	4

Potency and efficacy (% of quisqualate E_max_) of receptor internalization upon receptor stimulation or direct PKC stimulation obtained in four individual experiments performed in triplicate. –: variation is not available as quisqualate and was used for efficacy normalization.

### Generation of cPKC and nPKC KO HEK293A cell lines

**Figure 2:**
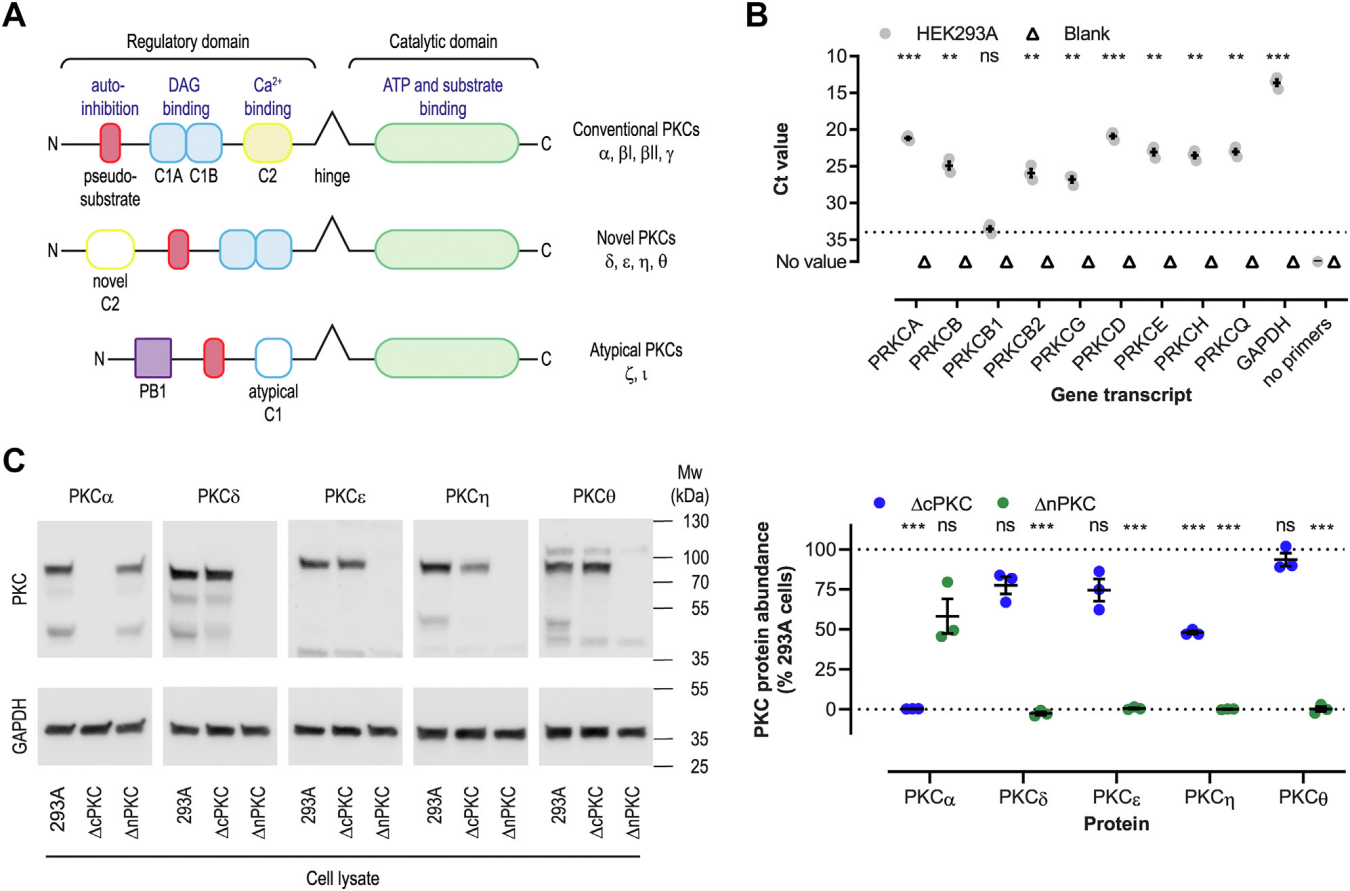
**Generation of PKC KO HEK293A cells.***A*, schematic representation of the structural differences between PKC subclasses. cPKC and nPKC are DAG sensors, whereas only cPKCs respond to Ca^2+^. aPKC isozymes are insensitive to these second messengers. *B*, qPCR-mediated evaluation of *PRKC* gene transcription in HEK293A cells. *C*, representative Western blot images and quantification of PKC protein levels normalized to GAPDH household protein in genetically modified HEK293A ΔcPKC and HEK293A ΔnPKC as compared to unmodified HEK293A cells. PKCβ and PKCγ protein could not be detected in HEK293A cells. Data represent mean and S.E.M. of three independent experiments performed in (*B*) duplicate or (*C*) singlicate. ns *p* ≥ 0.05, ∗∗*p* < 0.01, ∗∗∗*p* < 0.001, as determined using one sample t-tests when comparing to reference value 34 (B) and 100 (C). cPKC, conventional PKC; nPKC, novel PKC; aPKC, atypical PKC; DAG, diacylglycerol.

To investigate GPCR regulation by PKC in more detail, PKC KO cell lines were generated via CRISPR/Cas9-mediated gene editing. The human genome encodes eight second messenger–sensitive PKC isozymes, including two splice variants of the *PRKCB* gene product, which are activated upon PLC signaling by GPCRs (Fig. 2*A*). Quantitative PCR (qPCR) was used to determine *PRKC* gene transcription in HEK293A cells. qPCR reactions using selected primer pairs yielded between 96 and 104% efficiency for the target genes and thus were suited to evaluate gene transcription in cells (Fig. S2). All conventional and novel *PRKC* genes were transcribed in HEK293A cells, while splice variant *PRKCB2* was the only one of the two *PRKCB* gene products detected (Fig. 2*B*). Based on their differential mechanism of activation, we set out to generate two cell lines with full knockout of either cPKC or nPKC classes. Guide RNAs were designed to direct Cas9 to introduce double-strand DNA breaks upstream of the kinase domain and where possible downstream of the ATP-binding site, in *PRKC* genes. After clonal expansion, HEK293A clones containing frameshifts in all alleles of the targeted genes were identified using indel detection by amplicon analysis (40) (Table 2) and probed for PKC protein abundance. Endogenous PKCα, PKCδ, PKCε, PKCη, and PKCθ protein expression was shown in HEK293A cells. Even though overexpressed protein could be detected, endogenous PKCβI/II and PKCγ proteins were not detected in HEK293A cell lysates using several antibodies (see Experimental procedures, data not shown). HEK293A ΔcPKC cells were devoid of PKCα protein, and HEK293A ΔnPKC cells were depleted of PKCδ, PKCε, PKCη, and PKCθ proteins (Figs. 2*C* and S3). Compensatory increase in abundance of untargeted PKC proteins due to the loss of targeted PKC isozymes was not observed. Compared to WT cells, the PKCη protein level was approximately 50% reduced in HEK293A ΔcPKC cells. Selected clones appeared unchanged compared to parental HEK293A cells in terms of their morphology and growth.

**Table 2:** Frameshift mutations in gene-edited HEK293A cells

Clone	Gene	Mutation (bp)	Frequency (%)
ΔcPKC	*PRKCA*	+1	100
	*PRKCB*	−1	100
	*PRKCG*	−1	100
ΔnPKC	*PRKCD*	−2, +2	67, 33
	*PRKCE*	+1	100
	*PRKCH*	−10, +1	50, 50 or 33, 67∗
	*PRKCQ*	+1, +2	50, 50

Insertion (+) or deletion (−) of base pairs (bp) in *PRKC* genes. ∗HEK293A cells are polyploid cells with dynamic karyotype resulting in variable mutation frequency observed over time.

### Validation of a biosensor to monitor cellular PKC activity

A bioluminescence resonance energy transfer (BRET)-based PKC-c1b biosensor was employed to monitor cellular PKC activity (41). From N- to C-terminus, this membrane-anchored unimolecular sensor consists of a GFP10 protein, the forkhead-associated 1 (FHA1), and FHA2 phosphothreonine-binding domains derived from the Rad53 protein, a linker, two PKC substrate consensus sites, *Renilla* luciferase II (RlucII), and the DAG-binding motif c1b. Phosphorylation-driven recognition of the PKC substrate motifs by the phosphothreonine-binding domains brings RlucII and GFP10 in proximity, with concomitant increase in BRET signal.

**Figure 3:**
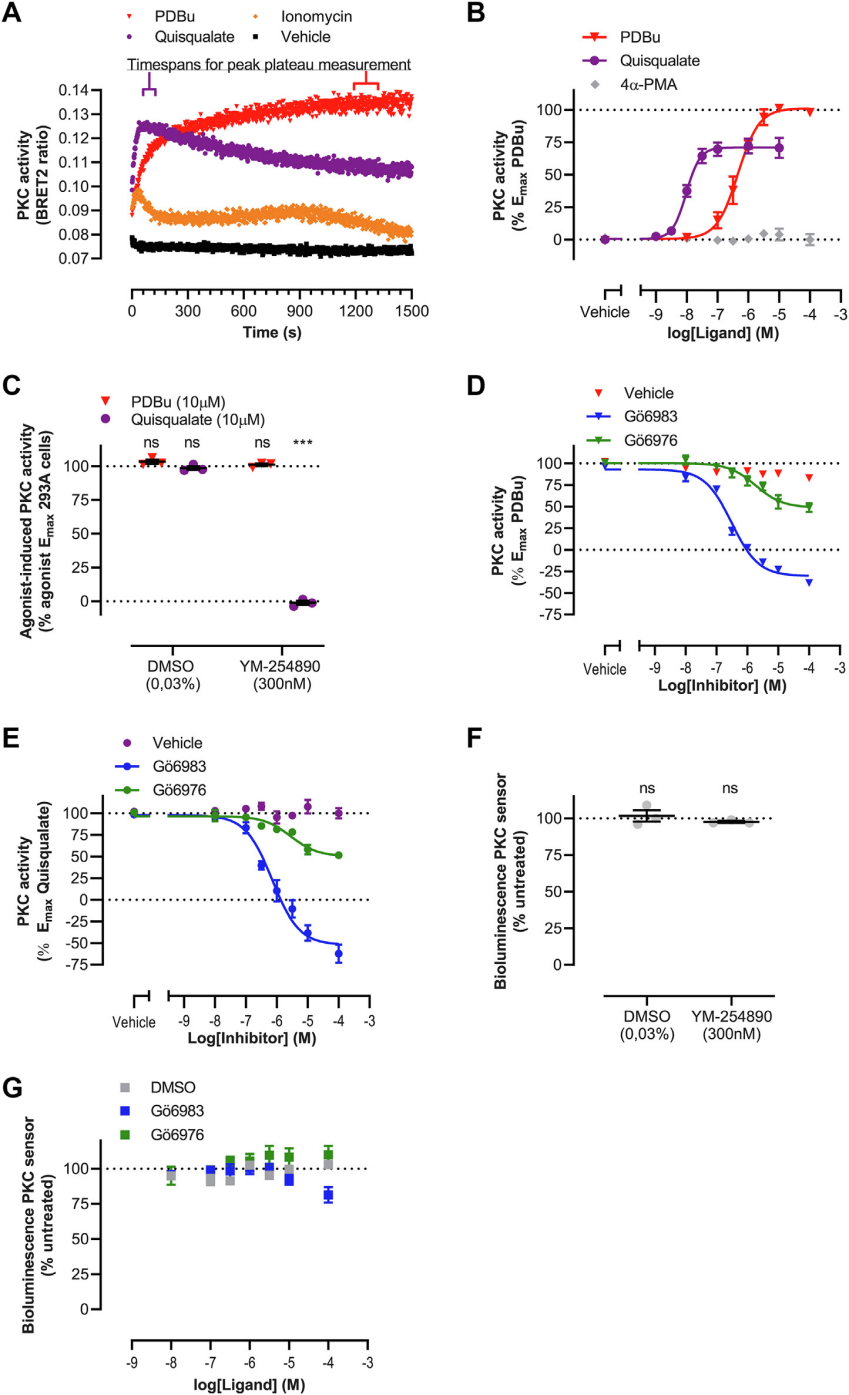
**Characterization of the PKC-c1b biosensor to evaluate cellular PKC activity.***A*, kinetic traces of PKC activation upon stimulation of mGlu_5a_-expressing HEK293A cells with PDBu, ionomycin, or quisqualate (10 μM each). Intervals selected for single point measurements of PDBu- and quisqualate-induced peak responses are indicated. *B*, concentration-dependent PKC activation in mGlu_5a_-expressing HEK293A cells by PDBu and quisqualate, but not 4α-PMA. Cellular responses were normalized to the E_max_ of PDBu. *C*, mGlu_5a_-expressing cells were treated with 300 nM YM-254890 for 30 min prior to stimulation with 10 μM PDBu or 10 μM quisqualate. Cellular responses were normalized to E_max_ of the respective agonist in vehicle-treated cells. mGlu_5a_-expressing cells were treated with concentration series of small molecule PKC inhibitors for 30 min prior to stimulation with 10 μM PDBu (*D*) or 10 μM quisqualate (*E*). Cellular responses were normalized to E_max_ of the respective agonist in vehicle-treated cells. Evaluation of the bioluminescence signal of the PKC biosensor upon treatment with (*F*) YM-254890 or (*G*) PKC inhibitors. Data show representative kinetic traces of three independent experiments performed in singlicate (*A*) or mean and S.E.M of three (*B*–*F*) or six (*B*, PDBu) independent experiments performed in duplicate (D + E + G) or triplicate (B + C + F). ns *p* ≥ 0.05, ∗∗∗ *p* < 0.001, as determined using one-way ANOVA with Holm-Sidak’s multiple comparison test on raw data with vehicle treated HEK293A cells stimulated with the same agonist as control condition. mGlu_5_, metabotropic glutamate receptor 5; PDBu, phorbol 12,13-dibutyrate.

Phospholipase Cβ activation by GPCRs, either via Gα_q/11_ or Gβγ proteins (9), leads to the production of inositol 1,4,5-trisphosphate and DAG, inositol 1,4,5-trisphosphate-mediated release of intracellular Ca^2+^ and subsequent second messenger–dependent activation of PKC (2). HEK293A cells expressing mGlu_5a_ receptor and the PKC biosensor were stimulated with either the β phorbol ester PDBu (mimicking DAG by binding to C1 domains), the α phorbol ester 4α-PMA (a negative control of PDBu, not binding to C1 domains), the calcium ionophore ionomycin, or the mGlu_5_ agonist quisqualate (Figs. 3*A* and S4). Ionomycin and quisqualate responses showed fast kinetics, with PKC activation peaking between 40 to 60 s and 40 to 120 s, respectively. PKC activation by PDBu was slower, peaking at 20 to 25 min, whereas 4α-PMA stimulation did not modulate PKC activity. Subsequent measurements of PKC activity upon PDBu and quisqualate stimulation were conducted during the plateau phase of their respective peak responses. The incubation time used for 4α-PMA was matched to that of PDBu. Concentration-dependent PKC activation was shown for PDBu and quisqualate, illustrating a more efficacious response upon direct PKC stimulation than mGlu_5a_-mediated Ca^2+^ and DAG signaling (Fig. 3*B* and Table 3). PKC activity was not affected by 4α-PMA at the concentration range evaluated (up to 100 μM). Saturating concentrations of PDBu and quisqualate (both 10 μM) were selected for follow-up experiments.

**Table 3:** PKC activity in mGlu_5a_-expressing HEK293A cells

Ligand	Quisqualate	PDBu
pEC_50_		
Mean	8.04	6.37
S.E.M.	0.06	0.10
E_max_ (%)		
Mean	69	100
S.E.M.	5	−

Potency and efficacy (% of PDBu E_max_) of PKC activation upon receptor stimulation or direct PKC stimulation obtained in three (quisqualate) or six (PDBu) individual experiments performed in triplicate. –: variation is not available as PDBu and was used for efficacy normalization.

The PKC activity sensor was further characterized using small molecule inhibitors of PKC and Gα_q/11_ proteins. Inhibition of Gα_q/11_ using YM-254890 fully prevented the quisqualate-induced receptor-mediated response, while direct activation of PKC by PDBu was unaffected by this inhibitor (Fig. 3*C*). cPKC inhibitor Gö6976 reduced both PDBu- and quisqualate-induced PKC activity by approximately 50% (Fig. 3, *D*, *E* and Table 4). Preincubation with the pan-PKC inhibitor Gö6983 completely inhibited PKC activation by PDBu and quisqualate and even reduced PKC activity below basal cellular level (Fig. 3, *D*, *E* and Table 4). The bioluminescence RlucII signal of the sensor was measured in presence of the inhibitors to evaluate the potential interference of the inhibitors with the assay readout. YM-254890 and Gö6976 showed no interference at the used concentrations, whereas Gö6983 reduced bioluminescence by 19% at the highest concentration evaluated (Fig. 3, *F* and *G*).

**Table 4:** Inhibition of cellular PKC activity by Gö6976 and Gö6983

Inhibitor	Gö6976		Gö6983	
Agonist	PDBu (10 µM)	Quisqualate (10 µM)	PDBu (10 µM)	Quisqualate (10 µM)
pIC_50_				
Mean	5.71	5.50	6.51	6.12
S.E.M.	0.36	0.11	0.08	0.10
Inhibition (%)				
Mean	53.1	46.8	123.9	151.1
S.E.M.	4.1	5.2	3.1	10.5

Potency and efficacy (% of E_max_ respective agonist) of PDBu- and quisqualate-induced PKC activation by Gö6976 and Gö6983 obtained in three experiments performed in duplicate.

**Figure 4:**
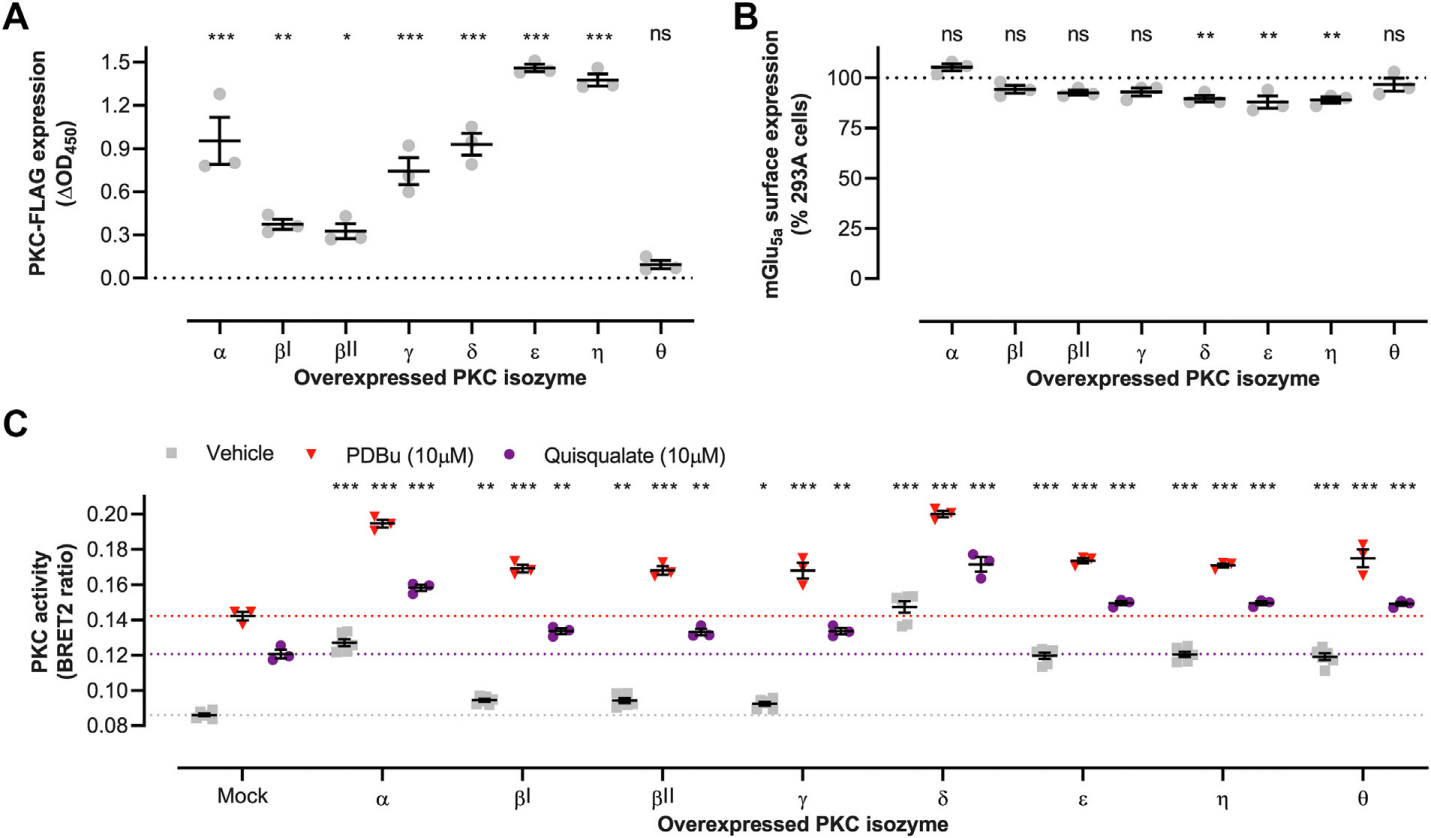
**Evaluating the sensitivity of the PKC-c1b biosensor to activation by individual PKC isozymes.** PKC-FLAG isozyme expression (*A*), HA-mGlu_5a_ expression (*B*), and PKC activity (*C*) in HEK293A cells cotransfected with individual PRKC genes and mGlu_5a_. The *gray*, *purple*, and *red dotted lines* indicate the level of basal-, quisqualate-, and PDBu-induced PKC activity in control HEK293A cells with endogenous PKC expression, respectively. Data represent mean and S.E.M of at three or six (*C*, vehicle stimulation) independent experiments performed in duplicate (*C*) or triplicate (A + B). ns *p* ≥ 0.05, ∗*p* < 0.05, ∗∗*p* < 0.01, ∗∗∗*p* < 0.001, as determined using one-way ANOVA (A + B) or two-way ANOVA (*C*), both with Holm-Sidak’s multiple comparison test, on raw data with mock transfected HEK293A cells stimulated with the same agonist as control condition. mGlu_5_, metabotropic glutamate receptor 5; PDBu, phorbol 12,13-dibutyrate.

Next, it was assayed whether the PKC substrate consensus sites in the biosensor (41, 42) are sensitive to phosphorylation by all second messenger–sensitive PKC isozymes. Cotransfection of equal amounts of plasmid DNA encoding individual PKC isozymes resulted in a wide range of PKC overexpression (Fig. 4*A*). PKC overexpression had limited effect on mGlu_5a_ receptor surface expression, with PKCδ, ε, and η reducing receptor expression by 12% or less compared to mock transfections (Fig. 4*B*). Elevated abundance of each PKC isozyme increased basal and/or PDBu- and quisqualate-induced BRET signals, illustrating that the biosensor is sensitive to phosphorylation by each cPKC and nPKC enzyme individually (Fig. 4*C*). Together, this demonstrates the PKC-c1b sensor is a valuable tool to monitor cellular PKC activity originating from all cPKC and nPKC isozymes.

### PKC activity is reduced in HEK293A ΔcPKC and ΔnPKC cells

**Figure 5:**
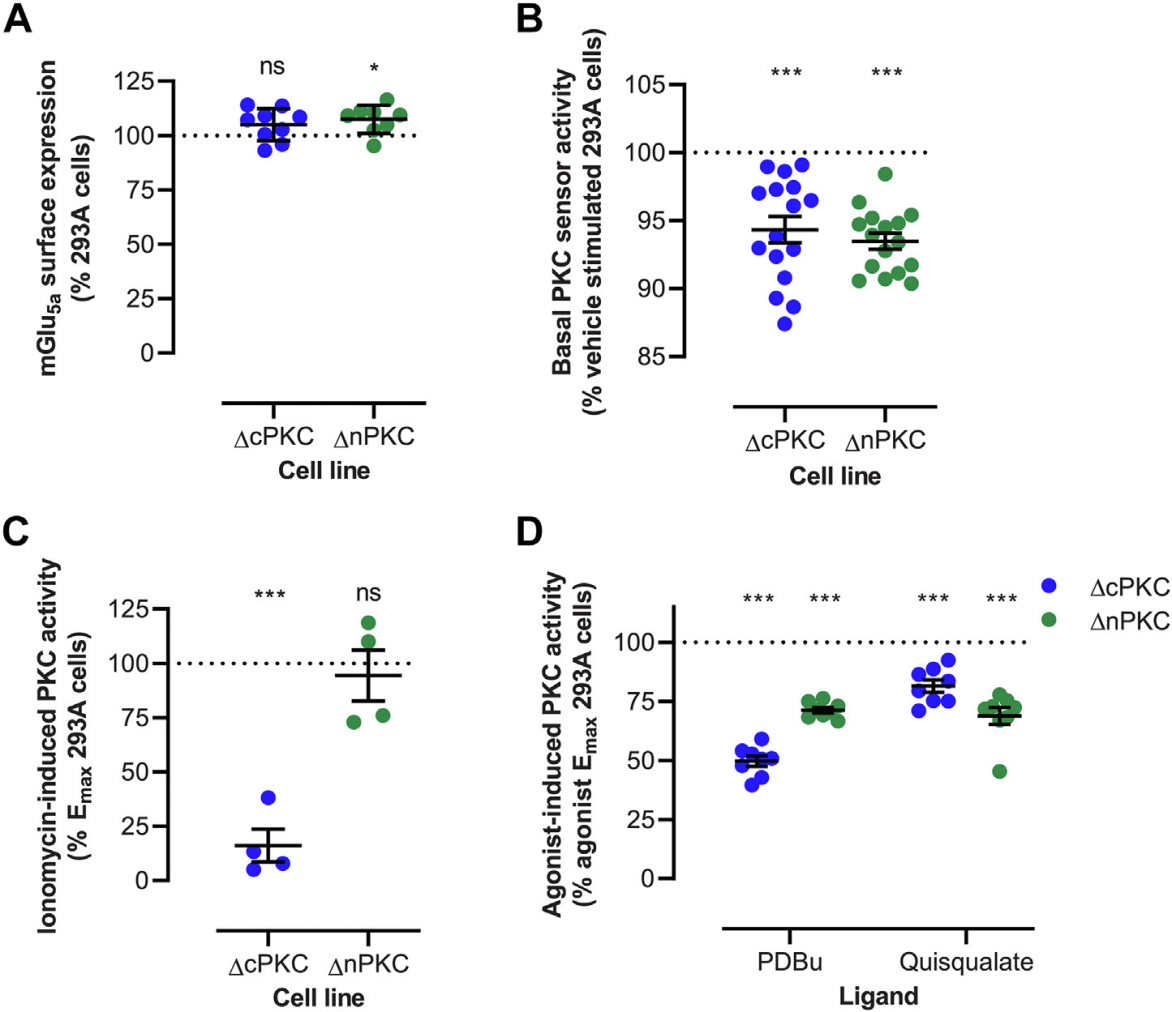
**PKC activity in HEK293A cells devoid of cPKC or nPKC.** Cellular PKC activity was monitored using the PKC-c1b sensor. mGlu_5a_ receptor expression (*A*), basal activity of the PKC sensor (*B*), and 10 μM ionomycin- (*C*), 10 μM PDBu-, and 10 μM quisqualate- (*D*) induced PKC activity in HEK293A ΔcPKC and HEK293A ΔnPKC cells, as compared to HEK293A cells. *C*, ionomycin-induced responses were determined by taking the area under the curve of 60-s real-time PKC activity traces after subtraction of corresponding buffer responses and were normalized to the ionomycin response in HEK293A cells. (*B* + *D*) basal, PDBu-, and quisqualate-induced responses were obtained by single point measurement at peak PKC activity, subtraction of corresponding buffer response and normalization to the E_max_ of the same agonist in HEK293A cells. Data represent mean and S.E.M of at least three independent experiments performed in triplicate. ns *p* ≥ 0.05, ∗*p* < 0.05, ∗∗∗*p* < 0.001, as determined using one-way ANOVA with Holm-Sidak’s multiple comparison test on raw data with HEK293A cells as control condition. mGlu_5_, metabotropic glutamate receptor 5; cPKC, conventional PKC; nPKC, novel PKC; PDBu, phorbol 12,13-dibutyrate.

The PKC activity in HEK293A ΔcPKC and ΔnPKC cells was evaluated using the PKC-c1b sensor. mGlu_5a_ receptor expression in HEK293A ΔnPKC cells was 7% increased compared to parental HEK293A cells, whereas this was not altered in HEK293A ΔcPKC cells (Fig. 5*A*). Basal reporter activity, comprising both basal cellular PKC activity (intramolecular energy transfer) and intermolecular energy transfer, was reduced by approximately 5% in both ΔcPKC and ΔnPKC cells as compared to parental cells (Fig. 5*B*). Due to the brief nature of the peak response of PKC activation induced by ionomycin (Fig. 3*A*), the area under the curve during 60 s poststimulation was determined as a measure of the ionomycin effect. Knockout of calcium-sensitive cPKCs lowered the ionomycin-induced response by approximately 84%, whereas the ionomycin response in ΔnPKC cells remained similar to parental HEK293A cells (Fig. 5*C*). PKC activity induced by PDBu and quisqualate were reduced in both knockout cell lines (Fig. 5*D*). Compared to parental cells, the PDBu response was decreased by 50% in ΔcPKC cells and 29% ΔnPKC cells. PKC activity induced by quisqualate stimulation of ΔcPKC cells and ΔnPKC cells lowered by 19% and 31% compared to HEK293A, respectively. Together, this confirms reduced PKC activity in HEK293A cells devoid of cPKC or nPKC isozymes.

### mGlu_5a_ internalization, but not Ca^2+^ signaling, is unaffected by loss of cPKC or nPKC

**Figure 6:**
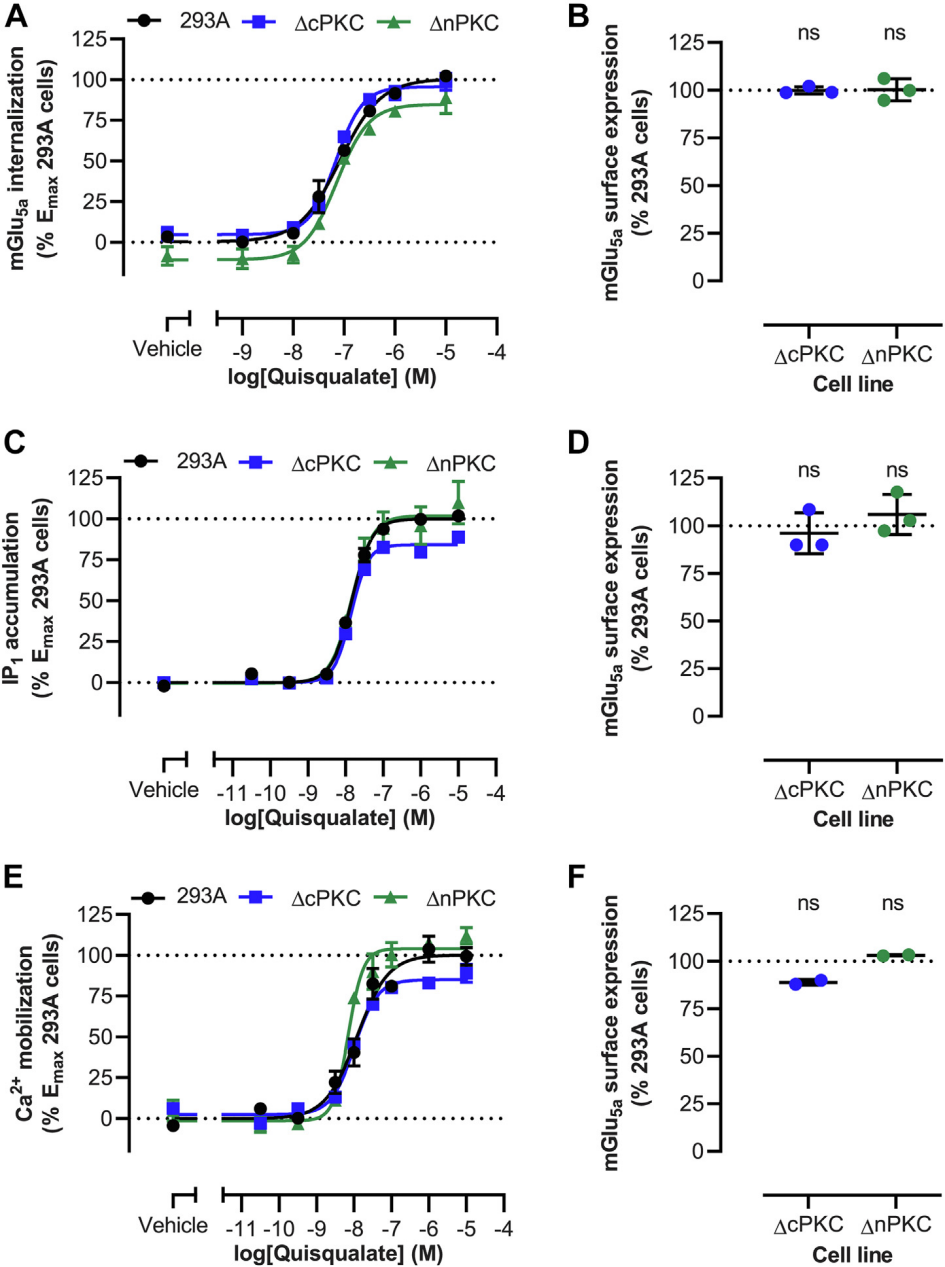
**mGlu**_**5a**_**internalization and signaling in parental, ΔcPKC, and ΔnPKC HEK293A cells.** Quisqualate-induced mGlu_5a_ internalization (*A*) and corresponding receptor expression (*B*) in HEK293A ΔcPKC and HEK293A ΔnPKC cells, as compared to HEK293A cells. Internalization was measured over a 120-min period following agonist addition. Agonists were present during the internalization measurements. Internalization was quantified by taking the area under the curve of the real-time internalization traces, subtraction of the buffer responses and normalization to the E_max_ obtained in HEK293A cells. Quisqualate-induced mGlu_5a_-mediated IP_1_ accumulation (*C*) and corresponding receptor expression (*D*) in HEK293A ΔcPKC and HEK293A ΔnPKC cells, as compared to HEK293A cells. Endpoint measurements were vehicle subtracted and normalized to the E_max_ obtained in HEK293A cells. Quisqualate-induced mGlu_5a_-mediated Ca^2+^ mobilization (*E*) and corresponding receptor expression (*F*) in HEK293A ΔcPKC and HEK293A ΔnPKC cells, as compared to HEK293A cells. Max ΔFluo-4 fluorescence signals were vehicle subtracted and normalized to the E_max_ obtained in HEK293A cells. Data represent mean and S.E.M of three (*A*–*D*) and two (E + F) independent experiments performed in triplicate. ns *p* ≥ 0.05, as determined using one-way ANOVA with Holm-Sidak’s multiple comparison test on raw data with HEK293A cells as control condition. cPKC, conventional PKC; IP_1,_ inositol monophosphate 1; nPKC, novel PKC; mGlu_5_, metabotropic glutamate receptor 5.

The contribution of cPKC and nPKC to mGlu_5a_ internalization and signaling was evaluated using the knockout cell lines at receptor cell surface expression levels similar to parental cells (Fig. 6, *B*, *D*, and *F*). Quisqualate-induced receptor internalization was not significantly affected in the absence of cPKC or nPKC in terms of potency and efficacy (Fig. 6*A* and Table 5). Upon quisqualate stimulation, mGlu_5a_-mediated inositol monophosphate (IP_1_) accumulation and Ca^2+^ mobilization in HEK293A ΔnPKC cells were both comparable to HEK293A cells (Fig. 6, *C*, *E* and Table 5). The efficacies of IP_1_ accumulation and Ca^2+^ mobilization were respectively reduced by 16% and 17% in the absence of cPKCs, of which only the change in Ca^2+^ mobilization was statistically significant. The potencies of the two quisqualate-induced responses were comparable in HEK293A ΔcPKC and HEK293A parental cells.

**Table 5:** mGlu_5a_ internalization and signaling in parental, ΔcPKC, and ΔnPKC HEK293A cells

Assay		Internalization			IP_1_ accumulation			Ca^2+^ mobilization		
Cell line		293A	ΔcPKC	ΔcPKC	293A	ΔcPKC	ΔcPKC	293A	ΔcPKC	ΔcPKC
pEC_50_	Mean	7.10	7.15	7.14	7.83	7.86	7.84	7.93	7.99	8.16
	S.E.M.	0.05	0.04	0.05	0.03	0.01	0.02	0.15	0.05	0.02
E_max_ (%)	Mean	100	91	96	100	84	102	100	83∗	105
	S.E.M.	−	2	12	−	3	12	−	2	3

Potency and efficacy (% of quisqualate response in HEK293A cells) of receptor internalization, IP_1_ accumulation, and Ca^2+^ mobilization induced by quisqualate obtained in respectively three, three, and two individual experiments performed in triplicate. ∗ *p* < 0.05, as determined using one-way ANOVA with Holm-Sidak’s multiple comparison test on raw data with HEK293A cells as control condition. –: variation is not available as quisqualate responses in HEK293A cells and were used for efficacy normalization.

## Discussion

**Figure 7:**
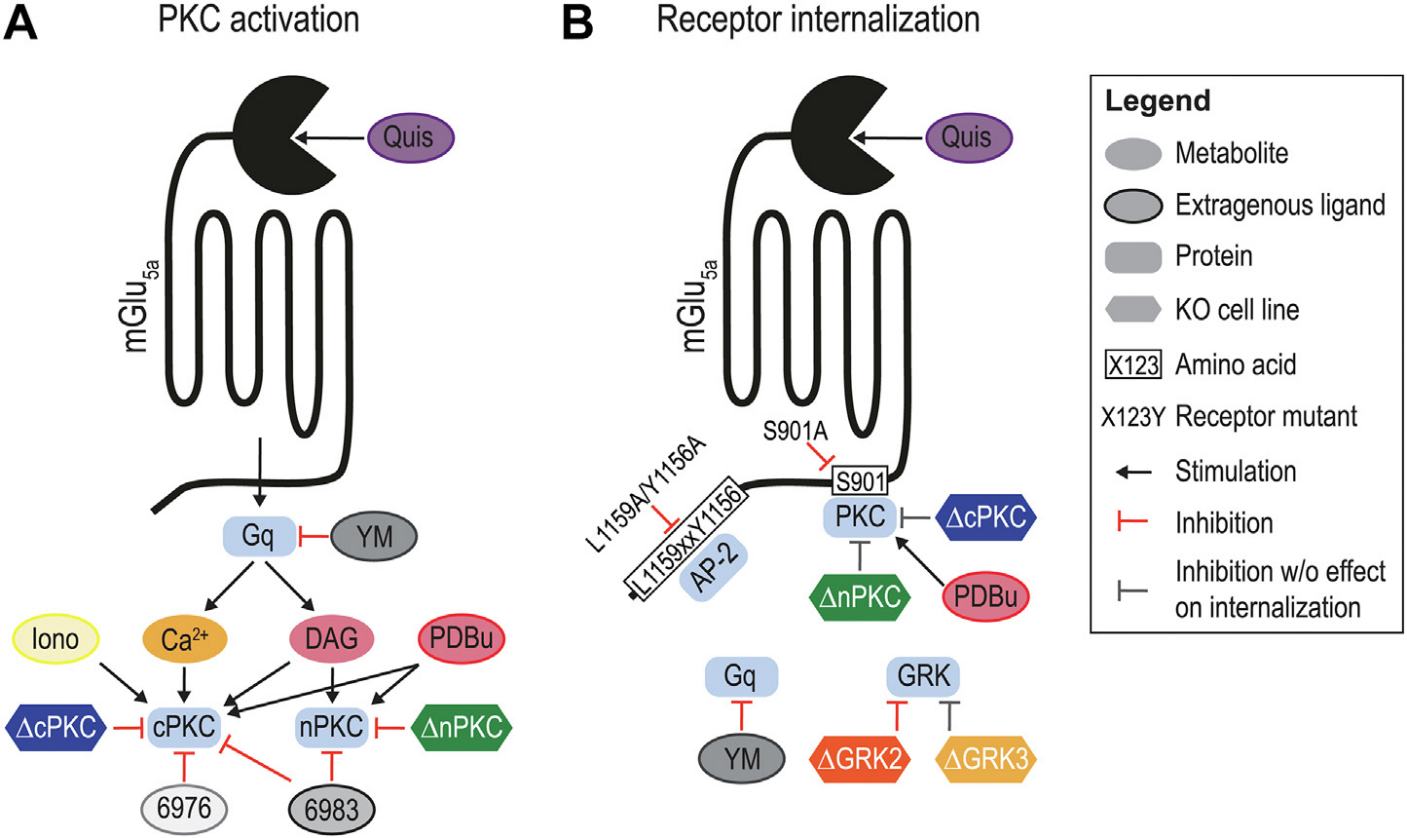
**Schematic overview of the characterization of mGlu**_**5a**_**-mediated PKC activation and mGlu**_**5a**_**internalization.** Research tools used to modulate cellular PKC activity (*A*) and mGlu_5a_ receptor internalization (*B*) in HEK293A cells. 6976; Gö6976, 6983; Gö6983, Iono; ionomycin, Quis; quisqualate, YM; YM-254890; mGlu_5_, metabotropic glutamate receptor 5.

In this study, we generated ΔcPKC and ΔnPKC cell lines derived from the commonly utilized HEK293A cellular background. These cell lines complement the toolbox comprising G protein- (10), GRK- (11), and arrestin-null (14) HEK293A cell lines available to study the role of these proteins in GPCR signaling and regulation. We employed the toolbox and additional approaches to dissect internalization of mGlu_5_ and show this to be a largely Gα_q/11_-dependent process, additionally driven by partial contributions of GRK2, PKC, and AP-2 (Fig. 7*B*). However, receptor internalization was not impacted by the absence of cPKCs or nPKCs, indicating involvement of isozymes from both subclasses that can compensate for each other. As such, mGlu_5_ internalization is another example of functional redundancy within the PKC family (Table S1) (43, 44, 45, 46, 47, 48).

In line with previous work (30, 31), we report that internalization of mGlu_5a_ can be stimulated through direct activation of DAG-sensitive PKC isozymes and that agonist-induced internalization is impaired upon mutation of PKC substrate residue Ser^901^ of the receptor. Both cPKC and nPKC isozymes seem to be involved, yet dispensable, for receptor internalization as mGlu_5a_ internalization was unchanged in cell lines devoid of cPKCs or nPKCs. PKCα is the only cPKC isozyme detected at protein level in HEK293A cells, implicating PKCα-mediated phosphorylation of mGlu_5_ at Ser^901^. We have not distinguished which nPKC isozymes facilitate mGlu_5_ internalization. Schwendt et al., however, have reported a role for PKCε (33). Hence, to determine the contribution of nPKC enzymes other than PKCε, it would be interesting to evaluate mGlu_5_ internalization in HEK293A cells devoid of both PKCα and PKCε and in cells depleted of all cPKC and nPKC in further studies. Since direct stimulation of PKCε translocation induced mGlu_5_ internalization to the same degree as receptor stimulation by surrogate agonist 3,5-dihydroxyphenylglycine in rat nucleus accumbens (33), it would also be informative to evaluate PKCα expression in this tissue. In our HEK293A model system, receptor endocytosis was only partially (about 40%) mediated by PKC, indicating additional cell type- or tissue-dependent mechanisms of mGlu_5_ regulation. Differences in PKC protein abundance, PKC- and/or mGlu_5_-interacting protein expression, alternative splicing of mGlu_5_, and quisqualate- or 3,5-dihydroxyphenylglycine–induced receptor signaling but also structural differences between human and rat PKC orthologs might explain observed differences. Alternatively, peptides modulating the translocation of PKC isozymes, used to show PKCε-mediated mGlu_5_ internalization (33), might be less specific than assumed (see below).

Based on their relative contributions obtained using the KO cell lines, cPKC and nPKC might not be the only contributors to PKC biosensor activation upon stimulation with PDBu and quisqualate (Fig. 7*A*). This might indicate that additional kinases, stimulated by PDBu and/or mGlu_5a_ signaling and inhibited by Gö6983, could activate the PKC-c1b biosensor. Alternatively, the loss of PKC isozymes might be compensated by the remaining PKC enzymes. However, protein abundance of the untargeted PKC isozymes was not increased. Furthermore, the ionomycin response in the ΔnPKC cell line was comparable to parental HEK293A cells, indicating that the activity of aPKCs in this cell line is not changed.

Although mGlu_5_ was previously reported to internalize in a clathrin-independent fashion based on the observation that its endocytosis was not affected by a dominant negative mutant of eps15 (49), reduced internalization upon mutation of the AP-2 interaction motif in mGlu_5_’s C-tail suggests participation in clathrin-mediated endocytosis. Indeed, more recent work has also shown redundancy of eps15 in this endocytosis pathway (50). Siah-1A–mediated receptor ubiquitination, which relies on PKC-mediated mGlu_5_ Ser^901^ phosphorylation for displacement of calmodulin, is important for mGlu_5_ internalization and degradation (31, 32). Yet, how ubiquitination of mGlu_5_ facilitates receptor endocytosis is still unknown. Epsins, a family of clathrin adaptor proteins, mediate internalization via clathrin-coated pits by binding ubiquitinated cargo through their ubiquitin-interacting motifs (51). These adaptors might therefore support endocytosis of ubiquitinated mGlu_5_, like has been shown for proteinase-activated receptor 1 (52).

Studies regarding the role of GRKs in mGlu_5_ regulation have thus far relied on overexpression experiments and shown that receptor desensitization is mediated by GRK2 and GRK3, but not GRK4 and GRK5 (53, 54, 55). Gα_q/11_ sequestration by GRK2 rather than enzymatic activity regulates inositol phosphate and Ca^2+^ signaling by this receptor (53, 54), whereas mGlu_5_-mediated GIRK channel activation is dependent on GRK2 kinase activity (55). The function of GRKs in receptor internalization is less clearly defined. Abreu et al. reported unchanged receptor internalization upon overexpression of GRK2 (53), while GRK2, independent of kinase activity, stimulated mGlu_5_ internalization in the hands of Ribeiro *et al.* (54). Using KO cell lines, we provide evidence that GRK2, but not GRK3, partially facilitates internalization of mGlu_5_.

Pharmacological compounds are commonly used to research the role of PKC in cell biology. The specificity of these tools, however, represents considerable shortcomings and results should therefore be interpreted with caution (26). PKC activation is typically achieved through elevation of intracellular Ca^2+^ or stimulation with DAG mimetics. CaMKII is an example of another Ca^2+^-regulated kinase with consensus phosphorylation sites similar to that of PKCs, signifying potential PKC-unspecific responses induced by this approach (24). Furthermore, β phorbol esters, such as PDBu, bind at least 18 C1 domain–containing DAG-sensitive proteins unrelated to PKC (26, 56). Small molecule inhibitors of PKC are frequently employed to determine the biological consequences of acute PKC inactivation but also show activity toward other kinases (26). In a screen against 300 kinases, Gö6983 and Gö6976, for instance, respectively inhibited the activity of 21 and 107 other kinases by more than 50%, underlining the promiscuity of these compounds (57). Peptides, based on the interaction interface between PKC and receptors for activated C kinases (RACKs), represent another type of PKC activators and inhibitors (58). RACKs are scaffolding proteins for PKC, anchoring enzymes in protein complexes, facilitating PKC isozyme localization, and stabilizing active conformations (25). Nonetheless, RACKs also bind other proteins via interaction motifs similar to PKC (58). This implicates potential disturbance of cellular localization of RACK-binding proteins other than PKC by these peptides. Depending on its protein abundance, PKCε furthermore associates with PKCβII-specific RACK1, exemplifying limited specificity (59). Driven by recent advances in genome editing techniques, PKC KO cell lines represent more specific research tools to study PKC-mediated aspects of cell biology. Hence, we welcome requests to obtain the ΔcPKC and ΔnPKC cell lines for future studies.

## Experimental procedures

### Materials

T4 poly nucleotide kinase and T4 ligase were purchased from New England Biolabs. Primers and oligos were purchased from TAG Copenhagen. PEI was purchased from Polysciences Inc. Gö6976 and Gö6983 were purchased from Selleck Chemicals and YM-254890 was bought from Tebu-bio. Coelenterazine 400a and ionomycin were purchased from Cayman Chemical Company. IP-One G_q_ kit and SNAP Lumi4-Tb labeling reagent were purchased from Cisbio. CoboXtract and PROFILase hot start DNA polymerase were purchased from COBO Technologies Aps. PDBu was purchased from Tocris Bioscience. PerfeCTa SYBR Green FastMix Low ROX, clear Falcon 96-well plates and black, clear-bottom Falcon 96-well plates were purchased from VWR. White, opaque CulturPlate-96 96-well plates and white, opaque OptiPlate-384 384-well plates were bought from PerkinElmer. 4α-PMA, fluorescein-O′-acetic acid, quisqualate, poly-D-lysine, RIPA buffer, cOmplete protease inhibitor cocktail, Trizma base, skim milk powder, Tween-X100, and peroxidase-conjugated anti-FLAG M2 (Sigma-Aldrich Cat# A8592, RRID:AB_439702) and peroxidase-conjugated anti-HA (Roche Cat# 12013819001, RRID:AB_390917) antibodies were purchased from Sigma-Aldrich. Mini-PROTEAN TDX Precast Protein Gels and Trans-Blot Turbo RTA Mini polyvinylidene difluoride Transfer Kit were purchased from Bio-Rad Laboratories. Dulbecco’s modified Eagle medium (DMEM, Cat# 61965059), dialyzed fetal bovine serum (dFBS), Opti-MEM, Penicillin-Streptomycin, Dulbecco’s phosphate buffered saline (DPBS, Cat# 14190-144), Hank’s balanced salt solution (HBSS, Cat# 14,175–053), Lipofectamine 2000 transfection reagent, 3,3ʹ,5,5ʹ - tetramethylbenzidine ELISA substrate, sulfuric acid, Fluo-4 acetoxymethyl cell permeant dye, probenecid, PureLink RNA mini Kit, PureLink DNase, High-capacity cDNA Reverse Transcription Kit, FastDigest BpiI, TOP10 *Escherichia coli.*, FastAP alkaline phosphatase, ATP, SYBR Safe DNA gel stain, Pierce BCA Protein Assay Kit, DTT, PageRuler Plus Prestained Protein Ladder (10–250 kDa), Pierce 10× Tris-Glycine SDS Buffer, NuPAGE LDS Sample buffer (4×), SuperSignal West Pico PLUS, and anti-PKCγ (Thermo Fisher Scientific Cat# PA5-28618, RRID:AB_2546094) and anti-mouse-AF800 (Thermo Fisher Scientific Cat# A32789, RRID:AB_2762832) antibodies were purchased from Thermo Fisher Scientific. Anti-PKCα (Abcam Cat# ab32376, RRID:AB_777294), anti-PKCδ (Abcam Cat# ab182126, RRID:AB_2892154), anti-PKCη (Abcam Cat# ab179524, RRID:AB_2892155), anti-PKCθ (Abcam Cat# ab110728, RRID:AB_10862201), and anti-PKCγ (Abcam Cat# ab71558, RRID:AB_1281066) antibodies were purchased from Abcam. Anti-PKCβ (Cell Signaling Technology Cat# 46809, RRID:AB_2799309) and anti-PKCε (Cell Signaling Technology Cat# 2683, RRID:AB_2171906) antibodies were bought from Cell Signal Technology. Anti-GAPDH (Novus Cat# NB600-502, RRID:AB_10077682) was acquired from Novus Biologicals. Anti-rabbit-HRP (Agilent Cat# P0448, RRID:AB_2617138) and anti-mouse-HRP (Agilent Cat# P0447, RRID:AB_2617137) antibodies were bought from Agilent. Anti-PKCβ (Santa Cruz Biotechnology Cat# sc-13149, RRID:AB_628144) and anti-PKCγ (Santa Cruz Biotechnology Cat# sc-166385, RRID:AB_2018059) antibodies were acquired from Santa Cruz Biotechnology. pcDNA3.1(+)/PRKCA-FLAG (CloneID OHu19098D), pcDNA3.1(+)/PRKCB1-FLAG (CloneID OHu17455D), pcDNA3.1(+)/PRKCB2-FLAG (CloneID OHu18081D), pcDNA3.1(+)/PRKCG-FLAG (CloneID OHu25732D), pcDNA3.1(+)/PRKCD-FLAG (CloneID OHu26841D), pcDNA3.1(+)/PRKCE-FLAG (CloneID OHu15997D), pcDNA3.1(+)/PRKCH-FLAG (CloneID OHu28046D), pcDNA3.1(+)/PRKCQ-FLAG (CloneID OHu20978D), pRK5/HA-SNAP-rmGlu_5a_-S901A, and pRK5/HA-SNAP-rmGlu_5a_-Y1156A + L1159A were purchased from GenScript. pSpCas9(BB)-2A-GFP was a gift from Feng Zhang (Addgene plasmid # 48138; RRID:Addgene_48138) (60). pRK5/hEAAT3 and pRK5/HA-SNAP-rmGlu_5a_ and pcDNA3.1(+)/PKC-c1b (41) were gifts from Jean-Philippe Pin and Laurent Prézeau and Michel Bouvier, respectively.

### Methods

#### Cell lines and culturing

The parental HEK293A cell line was provided by Dr Asuka Inoue. The HEK293A ΔGRK2 and HEK293A ΔGRK3 cell lines have been generated in our lab (11). All cell lines were cultured in pyruvate-free DMEM supplemented with 10% dFBS and 100 U/ml Penicillin-Streptomycin at 37 °C and 5% CO_2_ in a humidified incubator. Cell lines were tested negative for *mycoplasma* using mycoplasmacheck service by Eurofins Genomics.

#### RNA extraction and real-time qPCR

Total RNA was isolated from HEK293A cells using the PureLink RNA mini Kit, and genomic DNA was removed by on-column digestion using PureLink DNase, both according to manufacturer’s protocol. RNA concentration was determined using a NanoDrop2000 (Thermo Fischer Scientific), and cDNA was synthesized from 2 μg RNA using a High-Capacity cDNA Reverse Transcription kit, according to manufacturer’s protocol.

qPCR was performed on an Agilent M × 3005P qPCR System (Agilent Technologies) using 10 μl PerfeCTa SYBR Green FastMix, 5 μl five times diluted cDNA sample, and 6 pmol each of forward and reverse gene-specific primers (Table 6) in a 20 μl reaction volume. After initial denaturation at 95 °C for 30 s, 40 temperature cycles were conducted of 95 °C for 5 s, 60 °C for 15 s, and 72 °C for 10 s, followed by melt curve analysis. Efficiency of qPCR reactions was determined using standard curves of *PRKC*-encoding plasmid DNA (see materials section) and confirmed to be within the range of 90 to 110%, using the following formula: efficiency = 100∗((10^ˆ^(-1/slope))-1). Specificity was confirmed by single amplicon bands after agarose gel-electrophoresis.

**Table 6:** Primers used for qPCR

Locus	Primer	Sequence (5′-3′)	Amplicon size
*PRKCA*	Forward	TGCGGCAGAGATTTCCATCG	128
*PRKCA*	Reverse	TGCACATCCCAAAGTCAGCA	
*PRKCB*	Forward	AGGAGCCCCATGCTGTATTT	124
*PRKCB*	Reverse	ATGTGTCCCTCAGAATCGAGC	
*PRKCBI*	Forward	AGCCAAAAGCTAGAGACAAGAGA	83
*PRKCBI*	Reverse	ATCAGTGGGGGTCAGTTCCA	
*PRKCBII*	Forward	TGAACGCAAAGAGATCCAGC	
*PRKCBII*	Reverse	CTGGTCGGGAGGTGTTAGGA	112
*PRKCG*	Forward	CTACGCGGCAGAAATCGCTA	
*PRKCG*	Reverse	TGATGTGTCCCTCAGCATCC	107
*PRKCD*	Forward	GCCACATCAAGATTGCCGAC	
*PRKCD*	Reverse	GATATAGTCAGGGGTGCCGC	92
*PRKCE*	Forward	TGGATGCAGAAGGTCACTGC	
*PRKCE*	Reverse	GTCAGGAGTCCCACAGAACG	98
*PRKCH*	Forward	GGACCACGAGGGTCACTGTA	
*PRKCH*	Reverse	TGGAGCGATATAGTCTGGCG	109
*PRKCQ*	Forward	GGACACCTGACTACATCGCC	
*PRKCQ*	Reverse	GGCGACTGACCAATCAGCAT	112
*GAPDH*	Forward	GTTCGACAGTCAGCCGCATC	
*GAPDH*	Reverse	GCCCAATACGACCAAATCCGTT	106

#### Design and cloning of sgRNAs

Protospacer sequences were identified using the Custom Alt-R CRISPR-Cas9 guide RNA design tool from Integrated DNA Technologies. Exons of Homo Sapiens *PRKCA*, *PRKCB*, *PRKCG*, *PRKCD*, *PRKCE*, *PRKCH*, and *PRKCQ* genes were screened for protospacer adjacent motifs, preferably located upstream of the kinase active site sequence and downstream of the sequence encoding ATP-binding site of the kinases. Selected protospacer sequences (Table 7) contained at least three mismatches with predicted off-target sites and the list of off-target sites did not include obvious genes involved in GPCR biology. These protospacers were cloned into pSpCas9(BB)-2A-GFP plasmid (Addgene plasmid ID: 48138), as described previously (60), to create plasmids encoding PKC-specific sgRNAs, *Streptococcus pyogenes* Cas9 and GFP.

**Table 7:** sgRNAs used to target PRKC genes

Gene	RefSeq	Target exon	sgRNA	Protospacer (5′-3′)	Plasmid
*PRKCA*	NC_000017.11	10 (ENSE00003573265)	sgRNA-A	GTGCAGCTGCGTCAAGAACG	pX-A
*PRKCB*	NC_000016.10	9 (ENSE00001242785)	sgRNA-B	GACCGATTTTAACTTCCTAA	pX-B
*PRKCG*	NC_000019.10	6 (ENSE00001245692)	sgRNA-G	CTAATTCCTATGGACCCCAA	pX-G
*PRKCD*	NC_000003.12	11 (ENSE00001079761)	sgRNA-D	GAGCCTGTTGGGATATATCA	pX-D
*PRKCE*	NC_000002.12	11 (ENSE00003509073)	sgRNA-E	AATTCGACGAGCCTCGTTCA	pX-E
*PRKCH*	NC_000014.9	10 (ENSE00003468962)	sgRNA-H	AACGACGAGACTTCTGAATG	pX-H
*PRKCQ*	NC_000010.11	12 (ENSE00000689355)	sgRNA-Q	CGTCAGAAACGGATGCTCCC	pX-Q

PRKC gene and RefSeq, targeted exon and Ensembl exon ID, sgRNA name and protospacer sequence, and name of the plasmid after incorporation of the protospacer into pSpCas9(BB)-2A-GFP plasmid.

#### Generation and validation of CRISPR-Cas9 KO cell lines

The genetic modification approach was based on work by Lonowski et al (40). HEK293A cells and derived KO cells were seeded at a density of 5 × 10^5^ cells per 6-cm dish and incubated for 24 h at 37 °C and 5% CO_2_ in a humidified incubator. Using 7200 ng PEI, HEK293A cells were transfected with 600 ng each of pX-A and pX-G or pX-H and pX-Q, HEK293A ΔPKCαγ cells were transfected with 1200 ng of pX-B, and HEK293A ΔPKCηθ cells were transfected with 600 ng each of pX-D and pX-E. Two days posttransfection, the cells were reseeded in T25 flasks. On the third day posttransfection, the cells were harvested by trypsination and sorted based on their GFP expression using fluorescence-assisted cell sorting on a MoFlo Astrios Cell Sorter (Beckman Coulter). Single GFP-positive cells were sorted into wells of 96-well culture plates, containing culture media with 20% dFBS serum concentration to stimulate survival and growth of single cell clones. Plates were incubated at 37 °C and 5% CO_2_ in a humidified incubator for approximately 2 weeks and clonality was confirmed by visual inspection. Cells were subsequently expanded into 24- and 6-well plates when cell density of the colonies required to and pellets of 6 × 10^4^ cells were collected for genomic analysis. Upon expansion to 10-cm dishes, serum concentration in the culture media was reduced to 10% dFBS. Functional assays were performed after at least 3 weeks of cell culturing in culture medium containing 10% dFBS.

#### Indel detection by amplicon analysis

Cell pellets of single clones were dissolved in 30 μl CoboXtract followed by cell lysis (10 min at 70 °C, 10 min at 98 °C). A tri-primer touch-down PCR reaction was performed using PROFILase hot start DNA polymerase, according to manufacturer’s protocol, and primers listed in Table 8. Capillary electrophoresis-based fragment length analysis of the amplicons was performed by COBO Technologies Aps, and genetic modifications compared to control samples were determined using ProfileIT 1.0 software (COBO Technologies Aps).

**Table 8:** Primers used for IDAA of genetically modified HEK293A clones

Locus	Primer	Sequence (5′-3′)
*PRKCA*	Forward	AGCTGACCGGCAGCAAAATTG AGGATGATGACGTGGAGTGC
*PRKCA*	Reverse	GCATGAATGCCAAGCAGAGG
*PRKCB*	Forward	AGCTGACCGGCAGCAAAATTG ACCGGATGAAACTGACCGAT
*PRKCB*	Reverse	GACATTTTGCCCCAACACCT
*PRKCG*	Forward	AGCTGACCGGCAGCAAAATTG TTCCTGGATCTCTAACCCGTC
*PRKCG*	Reverse	GGTTCCCTCTATCCTAACCCC
*PRKCD*	Forward	AGCTGACCGGCAGCAAAATTG AGAGCCTCCCGGAGATCAG
*PRKCD*	Reverse	ACAGCCCGATTCACAGACTC
*PRKCE*	Forward	AGCTGACCGGCAGCAAAATTG TCTTCCACTAGACCGAGGTG
*PRKCE*	Reverse	GTTGCTAAGAGACGAGTGGGC
*PRKCH*	Forward	AGCTGACCGGCAGCAAAATTG TCGAGTCAAAGGTTTCTGATGC
*PRKCH*	Reverse	ATGGAGGAACATGAGAGCCG
*PRKCQ*	Forward	AGCTGACCGGCAGCAAAATTG TGATGTTGAGTGCACGATGG
*PRKCQ*	Reverse	CCTAAGTCACGGACTGCTGA
	FAM-Forward	6-FAM-AGCTGACCGGCAGCAAAATTG

#### Western blot

Lysate preparation of parental and genome-edited HEK293A cells and determination of lysate protein concentration was performed as previously described (11). Western blot samples were prepared with 38 μg protein in NuPAGE LDS Sample Buffer supplemented with 100 mM DTT and heated for 5 min at 95 °C. Subsequently, they were incubated for 15 min at room temperature and electrophoresed using 4 to 20% polyacrylamide gels for 40 min at 200 V. Proteins were transferred onto a PVDF membrane followed by 20 min blocking in TBS-T (10 mM Tris pH 7.4, 150 mM NaCl, 0.1% Tween 20) supplemented with 5% skim milk. Membranes were probed overnight on a roller band at 4 °C with rabbit anti-PKCα (1:4000), rabbit anti-PKCδ (1:4000), rabbit anti-PKCε (1:1000), rabbit anti-PKCη (1:2000), or rabbit anti-PKCθ (1:1000) antibodies, each combined with mouse anti-GAPDH (1:5000) in TBS-T/5% skim milk. The next day, membranes were washed three times for 10 min with TBS-T, blocked for 20 min with TBS-T/5% skim milk, and probed with goat anti-rabbit-HRP (1:2000) and donkey anti-mouse-AF800 (1:2000) antibodies in TBS-T/5% skim milk for 1 h at room temperature on a roller band. After two washes with TBS-T and one wash with TBS, blots were imaged for fluorescent signal and subsequently developed with chemiluminescent substrate and imaged for chemiluminescence on an iBright FL1500 (Thermo Fisher Scientific). Band intensities were quantified using Image Studio Lite software (LI-COR Biosciences).

Similarly, PKCβ and PKCλ abundance was probed in up to 150 μg HEK293A cell lysates using anti-PKCβ (Cell Signaling Technology Cat# 46809, RRID:AB_2799309, 1:1000 dilution; Santa Cruz Biotechnology Cat# sc-13149, RRID:AB_628144; 1:200 dilution) and anti-PKCγ (Abcam Cat# ab71558, RRID:AB_1281066, 1:2000 dilution; Santa Cruz Biotechnology Cat# sc-166385, RRID:AB_2018059, 1:100 dilution; Thermo Fisher Scientific Cat# PA5-28618, RRID:AB_2546094, 1:2000 dilution) antibodies, respectively. Primary antibodies of mouse origin were probed using anti-mouse-HRP antibody (1:2000 dilution), independent of GAPDH loading control probing. Lysates of HEK293A cells overexpressing PKCβ1/2 or PKCλ upon transfection with pcDNA3.1(+)/PRKCB1-FLAG, pcDNA3.1(+)/PRKCB2-FLAG, or pcDNA3.1(+)/PRKCG-FLAG were included as positive control samples to verify antibody performance.

#### Transfection conditions for functional assays and ELISA

Parental and genome-edited HEK293A cells were transiently transfected by reverse transfection of 5 × 10^4^ cells/well with 75 ng DNA and 01875 μl Lipofectamine 2000 in poly-d-lysine–coated 96-well plates or similar transfection conditions equivalent to 2 × 10^4^ cells/well in poly-d-lysine–coated 384-well plates. For internalization, IP-One, and Ca^2+^ mobilization assays with the mGlu_5a_ receptor, cells were transfected with 30 ng pRK5/HA-SNAP-rmGlu_5a_ and 5 ng pRK5/hEAAT3 supplemented to 75 ng DNA with pcDNA3.1(+) empty vector. When comparing rmGlu_5a_ with Ser^901^Ala and Tyr^1156^Ala/Lys^1159^Ala receptor mutants or HEK293A cells with HEK293A ΔGRK2, HEK293A ΔGRK3, or HEK293A ΔnPKC cells, 30 ng receptor-encoding plasmid DNA was used. To obtain similar receptor expression compared to HEK293A cells, HEK293A ΔcPKC cells were transfected with 60 ng instead of 30 ng pRK5/HA-SNAP-rmGlu_5a_ DNA. Compared to the other functional assays, cells were also transfected with 10 ng pcDNA3.1(+)/PKC-c1b BRET sensor for PKC activity experiments with the mGlu_5a_ receptor. In case of PKC overexpression for the PKC activity experiments, the abovementioned transfection mixture additionally contained 5 ng of pcDNA3.1(+)/PRKCA-FLAG, pcDNA3.1(+)/PRKCBI-FLAG, pcDNA3.1(+)/PRKCBII-FLAG, pcDNA3.1(+)/PRKCG-FLAG, pcDNA3.1(+)/PRKCD-FLAG, pcDNA3.1(+)/PRKCE-FLAG, pcDNA3.1(+)/PRKCH-FLAG, or pcDNA3.1(+)/PRKCQ-FLAG. For ELISA experiments, transfection conditions were identical to the matched functional assay. Functional assays were performed 24 h posttransfection.

#### Time-resolved Förster resonance energy transfer real-time internalization assay

On the day of the assay, culture media was removed from the white opaque CulturPlate-96 96-well plates or white opaque CELLSTAR 384-well plates and cells were incubated for 2 h at 37 °C in assay buffer (HBSS buffer supplemented with 20 mM Hepes, 1 mM MgCl_2,_ and 1 mM CaCl_2_ with pH adjusted to 7.4) followed by labeling of surface receptors with 100 nM SNAP Lumi4-Tb labeling reagent diluted in assay buffer supplemented with 0.1% bovine serum albumin for 1 h at 37 °C. After labeling, plates were washed once with assay buffer supplemented with 0.1% bovine serum albumin and twice with assay buffer. Donor fluorescence was obtained as a measure of receptor cell surface expression by excitation at 340/60 nm and emission measurement at 615/8.5 nm using an EnVision 2104 Multilabel Reader (PerkinElmer). Similarly, potential interference of compounds with donor fluorescence was evaluated. In case of inhibitor treatment, cells were preincubated with inhibitors for 30 min at 37 °C. Upon addition of fluorescein-O′-acetic acid (50 μM final concentration) and agonists, receptor internalization was measured using an EnVision 2104 Multilabel Reader every 3 min (96-well format) or every 6 min (384-well format) at 37 °C. After donor excitation at 340/60 nm, donor and acceptor emissions were measured at 615/8.5 nm and 520/8 nm, respectively. mGlu_5a_ receptor internalization was monitored for 66 or 120 min. Internalization ratios were calculated as 615 nm signal over 520 nm signal, and receptor internalization response was quantified as area under the curve of real-time internalization curves.

#### PKC activity assay

On the day of the assay, culture media was removed from the white opaque CulturPlate-96 96-well plates and cells were incubated for 2 h at 37 °C in assay buffer (HBSS buffer supplemented with 20 mM Hepes, 1 mM MgCl_2,_ and 1 mM CaCl_2_ with pH adjusted to 7.4). In case of inhibitor treatment, cells were preincubated with inhibitors for 30 min at room temperature prior to agonist stimulation. Potential inhibitor-mediated interference with the bioluminescence signal was evaluated in agonist-unstimulated cells and 1 s per well luminescence measurement at 410/80 nm, after 4 min preincubation with coelenterazine 400a (final concentration of 5 μM). To evaluate ligand-induced PKC activation kinetics over a time course of 25 min, cells were preincubated with coelenterazine 400a for 4 min, and one second interval kinetic-mode BRET^2^ measurements at room temperature were immediately initiated after agonist stimulation and mixing for 2 s. Due to its short-lived response, injectors were used to measure ionomycin-induced PKC activity in the different cell lines. Coelenterazine 400a was injected 10 s before ionomycin injection, each followed by 5 s of shaking. BRET^2^ was measured in kinetic-mode during 70 one second intervals at room temperature, and the area under the curve during the first 60-s poststimulation was used to quantify the ionomycin-induced response. For endpoint measurements, cells were preincubated with coelenterazine 400a and stimulated with quisqualate, 4α-PMA, or PDBu for 4-, 1-, 20-, and 20-min, respectively, prior to one second per well plate-mode BRET^2^ measurements at room temperature. BRET^2^ was measured using a LUMIstar Omega plate reader (BMG LABTECH) with 410/80 nm (donor) and 515/30 nm (acceptor) emission filters. BRET^2^ ratios were calculated as the ratio of acceptor over donor emission (515 nm/410 nm). For agonist-induced responses, the buffer response of the same cell line was subtracted to obtain ΔBRET ratios, which were then normalized to responses obtained in HEK293A cells.

#### IP-one assay

On the day of the assay, culture media was removed from the clear Costar 96-well plate and cells were incubated for 2 h at 37 °C in assay buffer (HBSS buffer supplemented with 20 mM Hepes, 1 mM MgCl_2,_ and 1 mM CaCl_2_ with pH adjusted to 7.4). After agonist stimulation for 1 h at 37 °C, cells were lysed with 30 μl lysis buffer (IP-One G_q_ kit) for 30 min at room temperature. Cell lysate and detection solution (IP-One G_q_ kit) were added (10 μl each) to a white, opaque OptiPlate 384-well plate and incubated at room temperature for 1 h in the dark. After excitation at 340 nm, emission was measured at 615 and 665 nm using an EnVision 2104 Multilabel Reader (PerkinElmer). FRET was calculated as 665 nm/615 nm ratios, converted into IP_1_ concentrations using an IP_1_ standard curve, and normalized to responses obtained in HEK293A cells.

#### Ca^2+^ mobilization assay

On the day of the assay, culture media was removed from the black-walled clear-bottom Falcon 96-well plate and cells were incubated for 2 h at 37 °C in assay buffer (HBSS buffer supplemented with 20 mM Hepes, 1 mM MgCl_2,_ and 1 mM CaCl_2_ with pH adjusted to 7.4). Next, cells were loaded with Fluo-4 acetoxymethyl cell permeant dye diluted in assay buffer supplemented with 2.5 mM probenecid for 1 h at 37 °C. Ligands were diluted in assay buffer supplemented with 2.5 mM probenecid and cells were preincubated with inhibitor BAPTA-AM for 20 min where indicated. Fluo-4 fluorescence (excitation at 485 nm and emission at 520 nm) was measured on a FlexStation 3 plate reader (Molecular Devices) at 37 °C for 17 s prior to and 120 s after stimulation with quisqualate. The maximum-minimum fluorescence was used as measure of Ca^2+^ mobilization.

#### ELISA

mGlu_5a_ receptor surface expression in PKC activity, IP-One, and Ca^2+^ mobilization experiments was determined by means of ELISA using horseradish peroxidase–conjugated anti-HA antibody (1:2000). PKC overexpression in PKC activity experiments was evaluated via ELISA using horseradish peroxidase-conjugated anti-FLAG M2 antibody (1:2000) in permeabilized cells. Cells were fixated in DPBS/4% paraformaldehyde for 5 min at room temperature. In case of permeabilization, cells were incubated in DPBS/0.5% Triton-X100 for 5 min at room temperature. Upon blocking with TBS-T/1% skim milk for 30 min at room temperature, cells were probed with above mentioned antibodies in TBS-T/1% skim milk for 1 h at room temperature. Wells were washed thrice with TBS-T and incubated in 3,3ʹ,5,5ʹ - tetramethylbenzidine for 4 min, before termination of the reaction using H_2_SO_4_. Absorbance was measured at 450 nm on an EnSpire microplate reader (PerkinElmer).

#### Data analysis and statistics

Results were analyzed using Prism 9 (GraphPad Software). Concentration-response curves were plotted using nonlinear regression curve fit with variable slope (four parameters). Statistical analyses were performed on raw data in which the data from the same individual experiment were paired (*t* test) or matched (ANOVAs) and the Holm-Sidak method was used to account for multiple comparisons. *p* < 0.05 was considered significant from the control condition. In Figure 2, *B* and *C*, where data was compared to a reference value without variance (34 ± 0 and 100 ± 0, respectively), observations were compared to the reference value using a one sample *t* test and the cutoff for what was considered a significant difference was adjusted to *p* < 0.05/n (n represents the number of compared observations) to account for multiple comparisons.

## Data availability

Data will be available upon request to the corresponding author.

## Supporting information

This article contains supporting information (43, 44, 45, 46, 47, 48).

## Conflict of interest

The authors declare that they have no conflicts of interest with the contents of this article.
