# Supplementary material for: Use of CRISPR/Cas9-edited HEK293 cells reveals that both conventional and novel protein kinase C isozymes are involved in mGlu5a receptor internalization
Source: J Biol Chem. 2022 Sep 8;298(10):102466. doi: 10.1016/j.jbc.2022.102466 (PMC9530845; doi:10.1016/j.jbc.2022.102466)
Supplement: supporting information [file mmc1.pdf]

Supporting Information

Title

Dissecting the roles of conventional or novel protein kinase C isozymes in $mGlu_{5a}$ receptor internalization using CRISPR/Cas9-edited HEK293 cells

Authors

Jeffrey R. van Senten, Thor C. Møller, Ee Von Moo, Sofie D. Seiersen and Hans Bräuner-Osborne.

Materials included ***page***

Figure S1.	S-2
Figure S2.	S-3
Figure S3.	S-4
Figure S4.	S-5
Table S1.	S-6
References	S-7

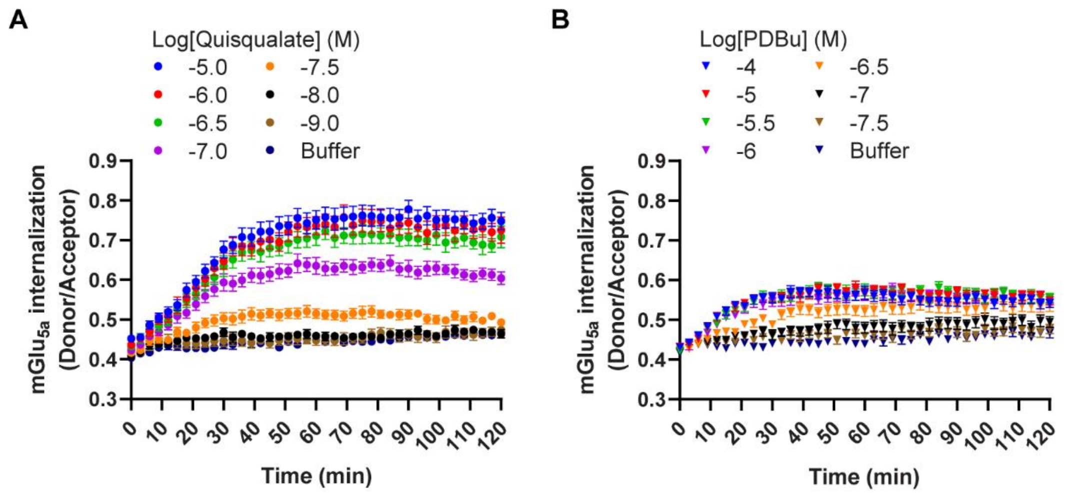

Figure S1. Real-time internalization of mGlu$_{5a}$. SNAP-tagged mGlu$_{5a}$ receptors at the cell surface of HEK293A cells were labeled with a FRET donor and cells were incubated in assay buffer containing a cell-impermeable FRET acceptor. Receptor internalization, shown as increase in the donor/acceptor ratio, upon stimulation with concentration series of quisqualate (A) or PDBu (B) was monitored in real-time at 37 °C. Data represent mean and S.E.M. of four independent experiments performed in triplicate.

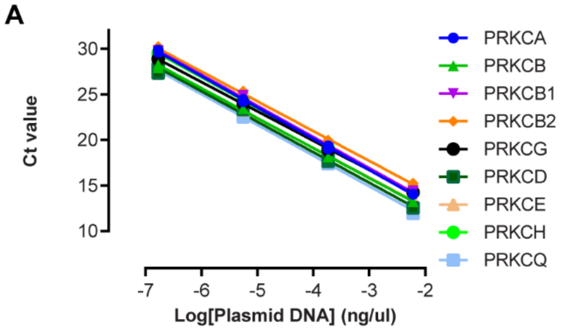

B

Target gene	Primer efficiency (%)
PRKCA	96
PRKCB	102
PRKCB1	96
PRKCB2	102
PRKCG	104
PRKCD	99
PRKCE	104
PRKCH	99
PRKCQ	98

Figure S2. Validation of qPCR primers used to determine *PRKC* gene transcripts. (A) Standard curves of *PRKC*-encoding plasmids to determine qPCR primer efficiency. **(B)** Efficiencies of qPCR reactions as determined in **(A)** and calculated using the formula; efficiency = 100*((10ˆ(-1/slope))-1). Data represent mean and SD of two independent experiments performed in duplicate.

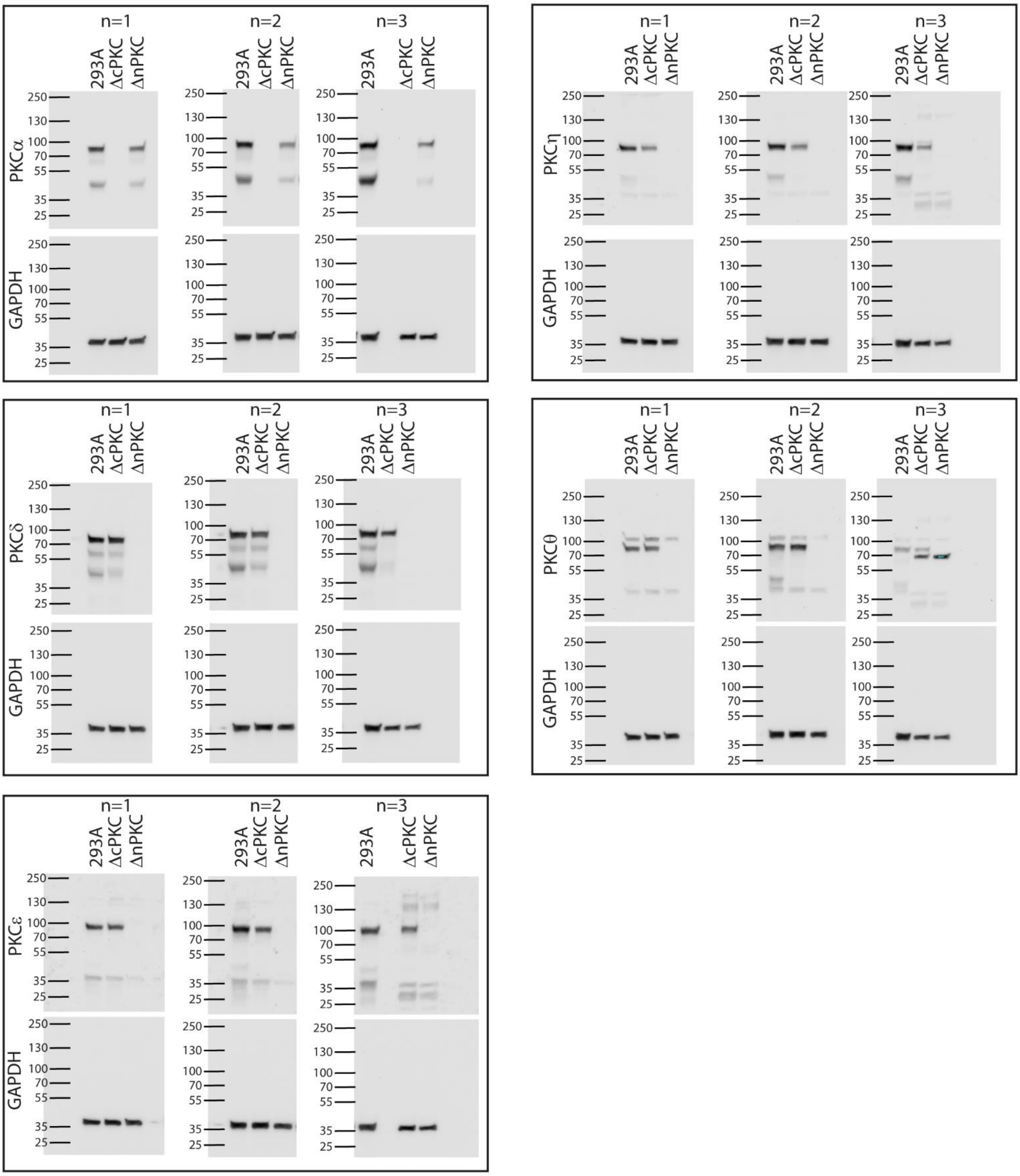

Figure S3. Individual full-length Western blot images used to determine PKCα, δ, ε, η and θ protein abundance in HEK293A, HEK293A ΔcPKC and HEK293A ΔnPKC cell lines. Representative Western blot images shown in Figure 2C of the main manuscript are the n=1 images for PKCα, δ, ε and the n=2 images for PKCη and θ.

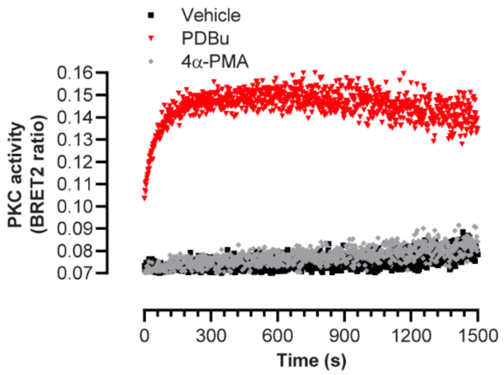

Figure S4. Characterization of the PKC-c1b biosensor to evaluate cellular PKC activity. Kinetic traces of PKC activity upon stimulation of mGlu$_{5a}$-expressing HEK293A cells with β phorbol ester PDBu or *α* phorbol ester 4*α*-PMA (10 μM each), obtained in a single experiment.

Table S1. Overview of functional redundancy within the PKC family.
[a] ortholog of mammalian PKCε. [b] ortholog of mammalian PKCη.

**PKC isozymes**	**Process**	**Evidence**
PKCα and PKCβ(1)	Stem cell factor-induced KIT receptor Y179 phosphorylation, p38 MAPK T180/Y182 phosphorylation, mouse bone marrow-derived mast cells viability and proliferation.	Inhibition upon double, but not individual, protein knock-down of PKCα and PKCβ.
PKCβ and PKCθ(2)	TGF-β activated kinase 1 activation in mouse T cells.	Inhibition upon double, but not individual, knock-out of PKCβ and PKCθ.
	IL-2 secretion by mouse T cells upon CD3 and CD28 stimulation.	Additive inhibition upon of double compared to individual knock-out of PKCβ and PKCθ.
PKCε and PKCη(3)	Promotion of PKCθ recruitment to the microtubule-organizing center of mouse T helper cells.	Inhibition upon double, but not individual, protein knock-down of PKCε and PKCη.
PKCη and PKCθ(4)	Mouse thymocyte development.	Poor development upon double, but not individual, PKCη and PKCθ knock-out in mice.
PKC-1[a] and TPA-1[b] (5)	Chemotaxis of *C. elegans* to NaCl.	Conditional compensation for the loss of PKC-1 by TPA-1 shown using individual and/or double knock-out of PKC-1 and TPA-1.
TTX-4[a] (PKC-1) and TPA-1[b] (6)	Chemotaxis of *C. elegans* to odorants.	Reduction in chemotaxis was rescued through PMA stimulation in individual, but not double, knock-out of TTX-4 and/or TPA-1.

References

1. Teegala, L. R., Elshoweikh, Y., Gudneppanavar, R., Thodeti, S., Pokhrel, S., Southard, E., Thodeti, C. K., and Paruchuri, S. (2022) Protein Kinase C alpha and beta compensate for each other to promote stem cell factor-mediated KIT phosphorylation, mast cell viability and proliferation. *FASEB J* **36**, e22273
2. Thuille, N., Wachowicz, K., Hermann-Kleiter, N., Kaminski, S., Fresser, F., Lutz-Nicoladoni, C., Leitges, M., Thome, M., Massoumi, R., and Baier, G. (2013) PKCtheta/beta and CYLD are antagonistic partners in the NFkappaB and NFAT transactivation pathways in primary mouse CD3+ T lymphocytes. *PLoS One* **8**, e53709
3. Quann, E. J., Liu, X., Altan-Bonnet, G., and Huse, M. (2011) A cascade of protein kinase C isozymes promotes cytoskeletal polarization in T cells. *Nat Immunol* **12**, 647-654
4. Fu, G., Hu, J., Niederberger-Magnenat, N., Rybakin, V., Casas, J., Yachi, P. P., Feldstein, S., Ma, B., Hoerter, J. A., Ampudia, J., Rigaud, S., Lambolez, F., Gavin, A. L., Sauer, K., Cheroutre, H., and Gascoigne, N. R. (2011) Protein kinase C eta is required for T cell activation and homeostatic proliferation. *Sci Signal* **4**, ra84
5. Hiroki, S., and Iino, Y. (2022) The redundancy and diversity between two novel PKC isotypes that regulate learning in Caenorhabditis elegans. *Proc Natl Acad Sci U S A* **119**
6. Okochi, Y., Kimura, K. D., Ohta, A., and Mori, I. (2005) Diverse regulation of sensory signaling by C. elegans nPKC-epsilon/eta TTX-4. *EMBO J* **24**, 2127-2137
